# Assimilation of Medical Appointment Scheduling Systems and Their Impact on the Accessibility of Primary Care: Mixed Methods Study

**DOI:** 10.2196/30485

**Published:** 2021-11-16

**Authors:** Guy Paré, Louis Raymond, Alexandre Castonguay, Antoine Grenier Ouimet, Marie-Claude Trudel

**Affiliations:** 1 Department of Information Technologies HEC Montréal Montréal, QC Canada; 2 École de gestion Université du Québec à Trois-Rivières Trois-Rivières, QC Canada; 3 Smith School of Business Queen's University Kingston, QC Canada

**Keywords:** medical appointment scheduling system, electronic booking, e-booking, primary care, accessibility of care, availability of care, advance access, electronic medical record

## Abstract

**Background:**

The COVID-19 pandemic has prompted the adoption of digital health technologies to maximize the accessibility of medical care in primary care settings. Medical appointment scheduling (MAS) systems are among the most essential technologies. Prior studies on MAS systems have taken either a user-oriented perspective, focusing on perceived outcomes such as patient satisfaction, or a technical perspective, focusing on optimizing medical scheduling algorithms. Less attention has been given to the extent to which family medicine practices have assimilated these systems into their daily operations and achieved impacts.

**Objective:**

This study aimed to fill this gap and provide answers to the following questions: (1) to what extent have primary care practices assimilated MAS systems into their daily operations? (2) what are the impacts of assimilating MAS systems on the accessibility and availability of primary care? and (3) what are the organizational and managerial factors associated with greater assimilation of MAS systems in family medicine clinics?

**Methods:**

A survey study targeting all family medicine clinics in Quebec, Canada, was conducted. The questionnaire was addressed to the individual responsible for managing medical schedules and appointments at these clinics. Following basic descriptive statistics, component-based structural equation modeling was used to empirically explore the causal paths implied in the conceptual framework. A cluster analysis was also performed to complement the causal analysis. As a final step, 6 experts in MAS systems were interviewed. Qualitative data were then coded and extracted using standard content analysis methods.

**Results:**

A total of 70 valid questionnaires were collected and analyzed. A large majority of the surveyed clinics had implemented MAS systems, with an average use of 1 or 2 functionalities, mainly “automated appointment confirmation and reminders” and “online appointment confirmation, modification, or cancellation by the patient.” More extensive use of MAS systems appears to contribute to improved availability of medical care in these clinics, notwithstanding the effect of their application of advanced access principles. Also, greater integration of MAS systems into the clinic’s electronic medical record system led to more extensive use. Our study further indicated that smaller clinics were less likely to undertake such integration and therefore showed less availability of medical care for their patients. Finally, our findings indicated that those clinics that showed a greater adoption rate and that used the provincial MAS system tended to be the highest-performing ones in terms of accessibility and availability of care.

**Conclusions:**

The main contribution of this study lies in the empirical demonstration that greater integration and assimilation of MAS systems in family medicine clinics lead to greater accessibility and availability of care for their patients and the general population. Valuable insight has also been provided on how to identify the clinics that would benefit most from such digital health solutions.

## Introduction

The accessibility of primary care services remains a global concern, and to address the underlying issues, considerable improvements must be made to health systems around the world [1,2]. Even well-developed universal health care systems that have been adopted by the most economically advanced and politically stable countries are not exempt from the need for major improvements [3].

Primary care clinics have adopted several organizational and technological solutions to maximize accessibility to care services. One such organizational solution used in primary care settings is called advanced access scheduling, a popular patient-centered scheduling model that enables patients to seek and receive medical care at a time that suits their needs and from the health care provider of their choice [4,5]. The main idea is to manage supply and demand efficiently by applying 5 key principles: balance supply and demand, reduce the backlog, review the appointment system, create contingency plans, and integrate interprofessional practices [6-9].

For their part, digital health technologies supporting medical appointment scheduling (MAS) represent innovations whose integration is critical for primary care clinics looking to improve accessibility [10-12]. Compelled to manage in-person and virtual consultations, family medicine practices face considerable challenges. MAS systems, also called electronic booking systems, are digital solutions that enable practices to streamline their management of medical scheduling through functionalities that optimize physician schedules according to preset parameters [13]. These systems also propose a convenient and accessible means for patients to manage their appointments with their care provider while facilitating online communication [14].

Health care authorities in some countries such as the United Kingdom and Canada have also sought to deploy interoperable medical appointment scheduling (iMAS) systems. Although MAS systems are proprietary, off-the-shelf software solutions that are customized to the needs of medical practices, iMAS systems are province-wide or country-wide platforms. Put simply, an iMAS can be defined as a one-stop, free, and centralized interface that allows individuals to book appointments at medical clinics of their choice. iMAS solutions extract data from proprietary MAS systems used in primary care clinics to display availabilities across clinics and regions. In Quebec, where this study was conducted, medical practices were encouraged to use the provincial iMAS system (Rendez-vous Santé Québec) instead of a proprietary MAS software solution. The deployment of centralized iMAS platforms aimed to increase the accessibility of medical care to the general population, including “orphan” patients who are not registered in any clinic, and to monitor the performance of primary care clinics regarding medical scheduling and appointments [15].

Considering the sizable impact of the COVID-19 pandemic on primary care and the well-known benefits of digital health tools to provide quality health care despite physical distancing, current data on the use of MAS systems and their impacts on access to family medicine care are essential. Prior empirical studies on this topic have mostly taken a user-oriented perspective, focusing on perceived patient outcomes such as satisfaction and individual impacts (eg, reduced waiting times) associated with various system features and user characteristics [16-19]. Further, MAS systems have mainly been studied from a technical perspective, focusing on optimizing medical scheduling algorithms [20,21]. However, less attention has been given to the extent to which family medicine practices have assimilated these systems and achieved impacts. Our study attempts to fill this gap. In particular, the assimilation of MAS systems is conceptualized as their integration into medical practices and extended use in their daily operations [22-24]. Moreover, the impacts of MAS assimilation are conceptualized as the accessibility and availability of medical care, a major dimension of organizational performance in family medicine settings [25]. In summary, this exploratory study aimed to provide answers to the following questions: (1) to what extent have primary care practices assimilated MAS systems into their daily operations? (2) what are the impacts of assimilating MAS systems on the accessibility and availability of primary care? and (3) what are the organizational and managerial factors associated with greater assimilation of MAS systems in family medicine clinics?

## Methods

This research was designed as a mixed methods study that included both a quantitative and a qualitative component. It received final approval from HEC Montréal’s ethics committee on March 20, 2020 (#2020-3784).

### Theoretical Background

**Figure 1:**
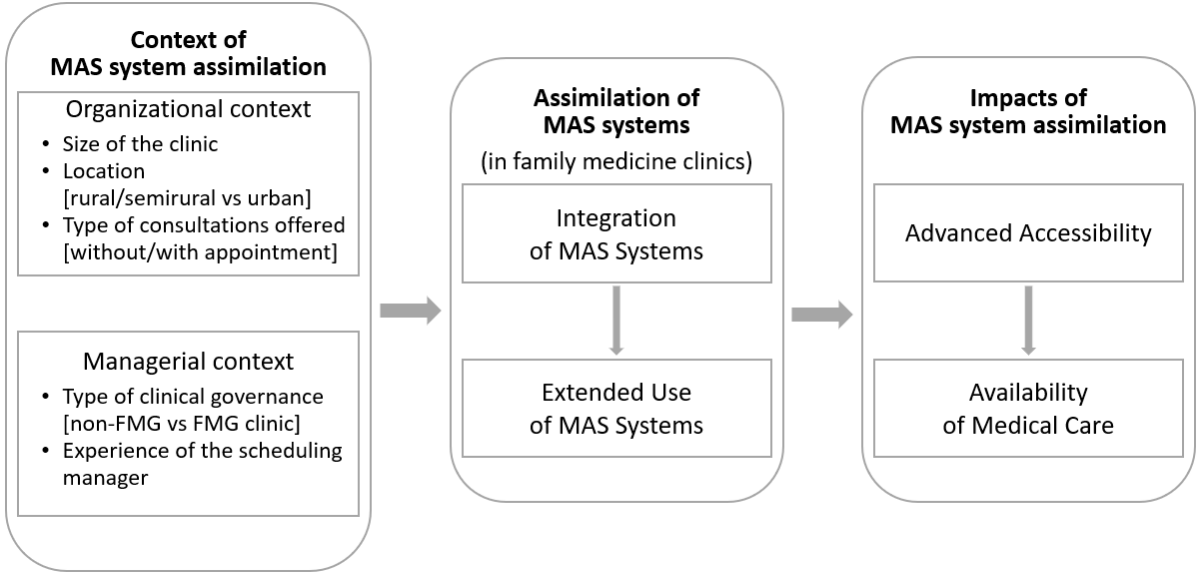
Conceptual framework. MAS: medical appointment scheduling, FMG: family medicine group.

In line with the abovementioned research questions, a conceptual framework was developed to describe and explain the assimilation of MAS systems in primary care clinics, as well as the potential influencing factors and performance outcomes of such an assimilation, as shown in Figure 1. The framework is based on previous works on the information technology (IT) assimilation theory. The basic tenet of the IT assimilation theory is that using IT-based systems per se does not necessarily lead to improved organizational performance [22]. Instead, an integrated, extended, and innovative use of IT systems are expected to positively impact the performance of health care organizations [23]. Thus, our initial research proposition is that the more integrated and extended the use of MAS systems, the greater their positive impact on family medicine clinics in terms of the accessibility and availability of medical care [24]. Moreover, following the IT assimilation theory, we propose that a more integrated use of MAS systems will lead to more extended usage in clinics [24]. Furthermore, the theory assumes organizational munificence and managerial readiness, in terms of IT and non-IT resources and competencies, to be critical influencing factors of IT assimilation, with organizational size and managerial experience being often employed in empirical studies as proxies [13,14]. We thus propose that the clinics’ organizational and managerial contexts will influence the integration and extended use of MAS systems. Importantly, based on previous work on organizational performance and advanced access scheduling in primary care settings [5], the last research proposition of our conceptual framework is that greater accessibility will lead to greater availability of medical care to the clinics’ patients and to the population at large.

Following the preceding theoretical propositions, the conceptual framework guided the design of the survey instrument administered to find answers to the research questions presented in Figure 1. Thus, the operationalization of the following 6 research constructs originates from extant literature on health IT assimilation in general and MAS systems in particular: organizational context [13,22,23], managerial context [13,24,26], integration of MAS systems [22,23], extended use of MAS systems [13,20,24], availability of medical care [13,17,21,25,26], and advanced accessibility [6-9,27].

### Mixed Methods Research Design

This study was primarily designed as a web-based survey. As described below, we followed best practices in web-based survey methodologies [28,29]. A pretest of the questionnaire was conducted at 5 primary care clinics, resulting in minor adjustments being made to the survey instrument. As a complementary qualitative approach, we interviewed 6 experts in MAS systems between February 12 and March 16, 2021. These experts include 2 physicians working full time in medical clinics, 1 family medicine group (FMG) administrator, 2 managers of the Quebec government health insurance system (Régie de l'Assurance Maladie du Québec), and 1 private MAS solution provider. Based on their extensive experience in Quebec’s medical sector, these respondents were asked to provide their interpretation of this study’s findings and evaluate whether these results were representative of the situation in medical clinics throughout the province. The web-based interviews were recorded and then transcribed. Qualitative data were coded and extracted using standard content analysis methods [30] and the NVivo software (version 1.4, QSR International). The experts’ insights were used mainly to enrich our discussion and interpretation of the survey findings.

### Sampling Procedure

The quantitative, survey-based component of our study targeted the 358 FMGs operating in Quebec, Canada. FMGs are primary care clinics structured and organized to provide Quebec’s population with greater accessibility to health care services. The basic tenets of FMG governance include teamwork and interdisciplinarity in the rendering of health care services to patients, registration of each patient to 1 family practitioner within the group for care and follow-up, and providing an option that allows registered patients to benefit from services that are integrated and offered nearby with extended office hours. Deployed on the Qualtrics web-based survey platform [31], the survey questionnaire was addressed to the individual responsible for managing medical schedules and appointments at these clinics. An invitation to participate in the study was sent on November 10, 2020, through a health care services newsletter whose distribution list comprises the FMGs in Quebec. The invitation contained a hyperlink directing the participants to the questionnaire through a secure website. This invitation was renewed 14 days later via the same newsletter.

### Statistical Analysis

Following basic descriptive statistics, component-based structural equation modeling (SEM) was used to empirically explore the causal paths implied in our conceptual framework. As implemented in SmartPLS software (version 2.0, SmartPLS GmBH), the partial least squares (PLS) SEM technique was chosen for its robustness regarding the distribution of residuals and its greater affinity for exploratory rather than confirmatory research purposes when compared to covariance-based SEM techniques [32]. A cluster analysis was also performed to complement the causal analysis.

## Results

### Sample Characteristics

A total of 70 valid questionnaires were collected from November 11 to December 20, 2020. Specifically, 60 were received from FMGs (17% response rate) and 10 from non-FMG clinics. Low response rates raise the possibility of nonresponse bias, so the potential for such bias was assessed by comparing the 47 “late” respondents (ie, those that responded after more than 14 days following the initial invitation) with the 23 “early” respondents [33]. An analysis of variance found no significant differences between these two sets of responding clinics in terms of the context and impacts of their assimilation of MAS systems, thus indicating a low probability of the presence of nonresponse bias in the study.

Of the 70 family medicine clinics sampled, 18 (26%) used a proprietary MAS software solution and Quebec’s iMAS provincial solution, 24 (36%) used only the iMAS system, 16 (23%) used only a proprietary MAS system, and 12 (17%) used no such system. At present, primary care clinics in Quebec are encouraged to use the iMAS system but are not obligated to do so, but this has not always been the case. Some FMGs have been obliged to use Quebec’s iMAS solution since its initial deployment. In the iMAS and MAS systems, the most used functionalities are “automated appointment confirmation and reminders” and “online appointment confirmation, modification, or cancellation by the patient.” The least used functionality was “invoicing compensatory fees for missed appointments.” Moreover, in the 42 clinical settings in which the iMAS system has been implemented, it was integrated into the clinics’ electronic medical record (EMR) system. Data transfers between the 2 systems were automated in most cases (n=38, 90%). Among the 34 clinics using the MAS system, 23 (67%) clinics had integrated it into their EMR system. The descriptive statistics of the research variables are presented in Tables 1 and 2.

We then characterized the 70 participating clinics based on the contexts and impacts of their assimilation of MAS systems. First, in terms of the organizational context, most (n=40, 57%) of the clinics were large (25,000 or more consultations per year), whereas small clinics (fewer than 5000 consultations per year) were the least represented in the sample (n=6, 8%). Many of the clinics (n=27, 39%) were located in rural, semirural, or remote regions. In terms of their openness to receiving “walk-in” patients, a significant proportion (n=16, 23%) of the clinics reported that half or more of their medical consultations were made without a prior appointment. Second, in terms of the managerial context, 86% (n=60) of the sampled clinics were found to be FMGs. Moreover, most (n=48, 69%) of the managers responsible for medical scheduling in the sampled clinics had 4 or more years of experience in their position.

More than half (n=36, 51%) of the 70 sampled clinics had either not implemented advanced access scheduling (14/70, 20%) or had done so in a tentative manner (22/70, 31%) by applying only 1 or 2 of its core principles (Table 2). In this regard, “rethinking the appointment system” by opening physicians’ working hours over a period of approximately 2 to 4 weeks was the only principle to have been applied by more than half of the sampled clinics (n=45, 64%). Approximately half of the sampled clinics reported making efforts to balance supply and demand as well as incorporate interprofessional practices (directing the patient to the right professionals according to their needs). Only a minority (n=11, 16%) of the clinics reported having prepared contingency plans for overflow periods.

The scheduling performance of the 70 clinics varied significantly, as 31% (n=22) scored themselves at ≤3 and 23% (n=16) at ≥4 on a 5-point scale, where 5 represents the highest performance level. On average, the sampled clinics considered their performance to be at its worst during enrollment of “orphan” patients, and their performance to be at its best when managing physician schedules. Overall, the respondents tended to think that their clinics performed at a rather high level. Moreover, the patient attendance rate was perceived to range from 80% to 89% for most of the sampled clinics (n=40, 57%), with a significant proportion (n=16, 23%) reaching an attendance rate of 90% or more.

**Table 1:** Descriptive statistics of the research variables (N=70).

Variable				Value
**Organizational context**				
	**Size of the clinic in terms of number of consultations, n (%)**			
		Less than 5000	6 (8)	
		5000 to 9999	11(16)	
		10,000 to 14,999	13 (19)	
		15,000 to 19,999	23 (33)	
		20,000 to 24,999	6 (8)	
		More than 25,000	11 (16)	
	**Location of the clinic, n (%)**			
		Nonurban (rural, semirural, or remote)	27 (39)	
		Urban	43 (61)	
	Type of consultations offered (without appointment), n (%)			20 (28)
**Managerial context**				
	**Type of clinical governance, n (%)**			
		FMG^a^	49 (70)	
		Non-FMG	21 (30)	
	**Experience of the scheduling manager, n (%)**			
		Less than 1 year	5 (7)	
		1 to 3 years	17 (25)	
		4 to 6 years	22 (31)	
		7 to 9 years	7 (10)	
		More than 10 years	19 (27)	
**Integration of MAS^b^ systems, n (%)**				
	**Clinic** **with implemented systems**			
		EMR^c^	69 (98)	
		iMAS^d^	42 (60)	
		iMAS integrated with the EMR	38 (54)	
		MAS	34 (49)	
		MAS integrated with the EMR	23 (33)	
		Integration of iMAS and MAS systems with the EMR	18 (26)	
**Extended use of MAS systems, mean (SD)**				
	iMAS system (RVSQ^e^) functionalities used^f^			0.8 (1.0)
	MAS system functionalities used^f^			1.6 (2.2)
**Advanced accessibility, mean (SD)**				
	Advanced access scheduling principles applied^f^			2.4 (1.8)
**Availability of medical care, mean (SD)**				
	Scheduling performance^g,h^			3.4 (0.7)
	Patient attendance^i^			1.6 (1.0)

^a^FMG: family medical group.

^b^MAS: medical appointment scheduling.

^c^EMR: electronic medical record.

^d^iMAS: interoperable medical appointment scheduling.

^e^RVSQ: Rendez-vous Santé Québec.

^f^See Table 2 for the distribution of this variable.

^g^Cronbach alpha coefficient of reliability (*α*=.76).

^h^1=totally disagree, 2=rather disagree, 3=neither disagree nor agree, 4=rather agree, and 5=totally agree.

^i^0=less than 80%, 1=80% to 84%, 2=85% to 89%, 3=90% to 94%, and 4=95% or more.

**Table 2:** Operationalization and distribution of the research variables (N=70).

Variable				Value
**iMAS^a^ system functionalities used, n (%)**				
	Automated appointment confirmation and reminder by email, SMS^b^ text messaging, or telephone			28 (67)
	Confirmation, modification, or cancellation of the appointment via the internet by the patient			21 (50)
	Invoicing compensatory fees for missed appointments			1 (2)
	Optimization of web-based appointment scheduling according to predetermined parameters (eg, adapted access, patient attendance, and avoidance of gaps in the schedule)			9 (21)
**MAS^c^ system functionalities used, n (%)**				
	Offer of appointments by automated telephone messages			20 (59)
	Automated appointment confirmation and reminder(s) by email, SMS text messaging, and telephone			22 (65)
	Confirmation, modification, or cancellation of the appointment via the internet by the patient			21 (62)
	Internet-based preconsultation questionnaire completed by the patient (reason for the consultation)			12 (35)
	Invoicing compensatory fees for missed appointments			5 (15)
	Optimization of web-based appointment scheduling according to predetermined parameters (eg, advanced access, attendance, avoidance of gaps in the schedule, and automated appointment scheduling for patients on the waiting list)			10 (29)
	Restriction of the appointment offer for certain patients (registered vs unregistered)			20 (59)
**Advanced access scheduling principles applied, n (%)**				
	Balancing supply and demand			34 (49)
	Reducing accumulated backlog^d^			27 (39)
	Rethinking the appointment system^e^			45 (64)
	**Developing contingency plans**			
		Schedule planning based on absences	18 (26)	
		Planning for overflow periods	11 (16)	
	Incorporating interprofessional practice			32 (46)
**Scheduling performance^f^, mean (SD)**				
	The number of missed appointments (“no-shows”) at my clinic is not a problem			3.4 (1.1)
	My clinic is still enrolling a large number of “orphan” patients			3.0 (1.3)
	The management of schedules by the administrative staff is very efficient			3.7 (1.0)
	Web-based appointment booking by the administrative staff is very efficient			3.3 (1.2)
	The satisfaction of the administrative staff in my clinic with regard to scheduling and making appointments is very high			3.3 (1.1)
	The satisfaction of the doctors in my clinic with regard to scheduling and making appointments is very high			3.6 (1.0)
	Patient satisfaction in my clinic with the way appointment scheduling works is very high			3.3 (1.1)
	Registered patients may obtain a consultation at my clinic within a very short time			3.6 (1.2)
	Unregistered patients may obtain a consultation at my clinic within a very short time			3.3 (1.3)

^a^iMAS: interoperable medical appointment scheduling.

^b^SMS: short message service.

^c^MAS: medical appointment scheduling.

^d^Using patient empowerment (eg, patient confirmation of appointment, missed appointment fee).

^e^Opening hours over a period of approximately 2 to 4 weeks.

^f^1=totally disagree, 2=rather disagree, 3=neither disagree nor agree, 4=rather agree, and 5=totally agree.

### Causal Analysis

In this study, all 6 research constructs were modeled as being “formative” given their composite and multidimensional nature (Figure 1). The first step in the data analysis consists of simultaneously estimating the measurement and theoretical models using the PLS SEM technique [32].

### Assessment of the Measurement Model

The metric properties of the research constructs were assessed within the context of the theoretical model. As the standard reliability and validity criteria applicable to reflective constructs do not apply to formative constructs, one must first verify that there is no collinearity among the formative construct’s indicators. To do so, the variance inflation factor (VIF) statistic is used, the rule being that the VIF must not be greater than 3.3 [34]. As presented in Table 3, the VIF values for all 11 indicators (research variables) were below this threshold (ranging between 1.00 and 1.12), confirming the absence of any multicollinearity. Once the validity of the measures has been assessed, the last property to be verified is discriminant validity, which shows the extent to which each research construct, as measured, is unique and different from the other five. This validity was confirmed by the fact that each construct shared less than 50% of its variance with any other construct (an interconstruct correlation of >0.7), as shown in Table 3.

**Table 3:** Validity of the research constructs^a^.

Research construct	Construct indicators^b^			Interconstruct correlation matrix				
	VIF_1_	VIF_2_	VIF_3_	1	2	3	4	5
1. Organizational context	1.11	1.12	1.02	—^c^	—	—	—	—
2. Integration of MAS^d^ systems	1.00	—	—	0.46	—	—	—	—
3. Managerial context	1.00	1.00	—	–0.63	–0.44	—	—	—
4. Extended use of MAS systems	1.05	1.05	—	0.51	0.64	–0.38	—	—
5. Advanced accessibility	1.00	—	—	0.19	0.29	–0.18	0.14	—
6. Availability of medical care	1.00	1.00	—	0.54	0.21	–0.35	0.26	0.39

^a^Assessing composite reliability and average variance extracted is inappropriate for formative constructs.

^b^VIF_i_: variance inflation factor of the construct’s *i*th indicator.

^c^Not applicable.

^d^MAS: medical appointment scheduling.

### Assessment of the Theoretical Model

**Figure 2:**
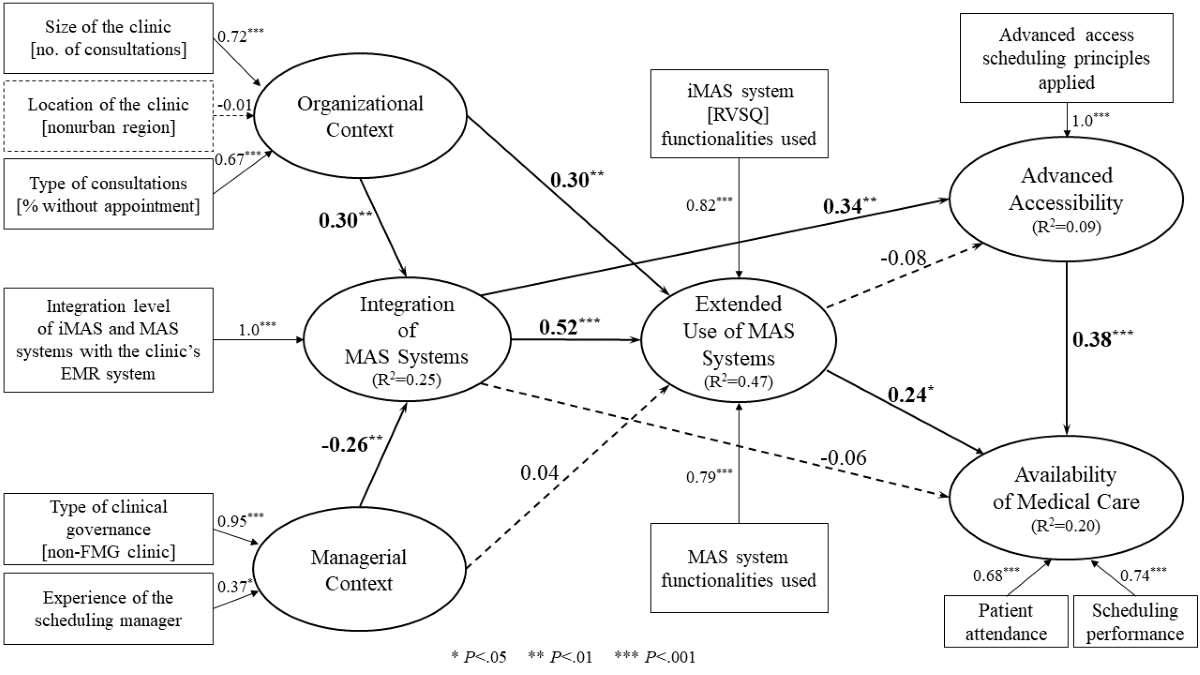
Results of the causal analysis. iMAS: interoperable medical appointment scheduling, MAS: medical appointment scheduling, EMR: electronic medical record, FMG: family medicine group, RVSQ: Rendez-vous Santé Québec.

As shown in Figure 2, the relationships inferred from the conceptual framework were tested by assessing the path coefficients (*β*) estimated by the SEM procedure, as executed by SmartPLS. The performance of the theoretical model highlighting interrelationships among the 6 research constructs was assessed by the strength and significance of the path coefficients (*β*) and the proportion of explained variance (*R*^2^), befitting the focus of the PLS method on the prediction and generalization [34].

Given the results of the causal analysis provided by the SEM procedure, the initial finding concerned the positive and significant path coefficients linking the sampled clinics’ organizational context to their integration (*β*=.3, *P*=.009) and extended use (*β*=.3, *P*=.009) of MAS systems. Hence, family medicine clinics that were larger in size and more open to walk-in patients showed greater assimilation of MAS technology in their daily operations. The second finding was that differences in the managerial context in terms of the clinics’ governance and the level of experience of their scheduling manager directly influenced their integration of MAS systems with their EMR systems (*β*=.26, *P*=.006). Here, the data showed that clinics whose governance was not of the FMG type and whose scheduling manager was more experienced had a lower level of system integration. Although the managerial context was found to have no direct effect on the extended use of MAS systems (*β*=.04, *P*=.38), it was nevertheless shown to have an indirect effect [35] through the mediating effect of MAS system integration.

Our results also suggested that the main precursor to the extended use of MAS systems is the sampled clinics’ integration of these systems with their EMR systems (*β*=.52, *P*<.001). Moreover, their integration of MAS systems with their EMR systems was also found to have a positive impact on their use of advanced access scheduling principles (*β*=.34, *P*=.005). Greater system integration appears to enable increased application of advanced access scheduling principles by these clinics, such as interprofessional work and joint monitoring. Although integration had no direct effect on the availability of medical care (*P*=.39), it did have significant indirect effects through the mediating impacts of advanced accessibility and extended use of MAS systems.

A positive and significant path coefficient was found between the use of MAS systems by family medicine clinics and the availability of medical care in these clinics (*β*=.24, *P*=.047). In contrast with the effect associated with the integration of MAS systems, the clinics’ use of MAS system functionalities appeared to have no significant impact on the extent to which they implemented advanced access scheduling principles (*P*=.28). One additional finding on the impacts of MAS system assimilation in family medicine clinics highlighted the role of advanced access scheduling as a precondition to increased availability of medical care in these clinics (*β*=.38, *P*<.001).

During causal analysis, we found significant and important indirect effects of the organizational context on the extended use of MAS systems (*P*=.03, 34% of total effects), namely through the integration of MAS systems, as shown in Table 4. There were also important indirect effects of integration on the availability of medical care (*P*=.10) through extended use. These findings highlight the “mediating” role played by the clinics’ assimilation of MAS systems in improving organizational performance in terms of medical care accessibility and availability.

**Table 4:** Breakdown of the total effects of the research constructs.

Relationship between the research constructs	Direct effects	Indirect effects	Total effects
Organizational context → Integration of MAS^a^ systems	0.295	0.000	0.295
Managerial context **→** Integration of MAS systems	–0.257	0.000	–0.257
Organizational context **→** Extended use of MAS systems	0.303	0.153	0.456
Managerial context **→** Extended use of MAS systems	0.041	–0.134	–0.093
Integration of MAS systems **→** Extended use of MAS systems	0.520	0.000	0.520
Organizational context **→** Advanced accessibility	0.000	0.064	0.064
Managerial context **→** Advanced accessibility	0.000	–0.081	–0.081
Integration of MAS systems **→** Advanced accessibility	0.258	0.042	0.300
Extended use of MAS systems **→** Advanced accessibility	–0.080	0.000	–0.080
Organizational context **→** Availability of medical care	0.000	0.114	0.114
Managerial context **→** Availability of medical care	0.000	–0.037	–0.037
Integration of MAS systems **→** Availability of medical care	–0.057	0.235	0.178
Extended use of MAS systems **→** Availability of medical care	0.235	–0.030	0.205
Advanced accessibility **→** Availability of medical care	0.377	0.000	0.377

^a^MAS: medical appointment scheduling.

### Cluster Analysis

To further explain the assimilation of MAS systems into primary settings, we applied an alternative approach to analyze our survey data. We followed a “case-oriented” approach as a complement to the preceding “variable-oriented” approach [36]. The case-oriented or configurational approach makes no assumptions about the statistical distribution of the research variables and the linearity of the relationships between these variables [37]. As it is operationalized with methods such as cluster analysis, this approach is meant to provide all-encompassing and holistic views regarding the use of MAS systems by family medicine clinics. It allows us to verify whether these clinics are better represented as a whole or as members of distinct subgroups, and to explore why and in what regard. A cluster analysis was thus conducted to group the surveyed clinics based on profiles that characterize the impacts of their assimilation of MAS systems on the accessibility and availability of medical care. A 3-cluster solution was found to be the most interpretable and meaningful one in terms of identifying profiles that could be clearly distinguished from one another. Given the exploratory research nature of this study and the goal of finding unknown groups (ie, groups not explicitly labeled in the data), the k-means clustering algorithm was used (SPSS Quick Cluster procedure) [38].

As shown in Table 5, among the 70 clinics, 15 (21%) clinics in the first profile were named “low performance” or “low” clinics (low levels of accessibility and availability). A second group of 25 (36%) clinics was named “mixed performance” or “mixed” (a low level of accessibility but a high level of availability). The third profile consisted of 30 (43%) clinics that were named “high performance” or “high” (high levels of accessibility and availability).

**Table 5:** Descriptive statistics and analysis of variance results for clinic profiles (N=70).

Research construct		Clinic profiles^a^			*F* value	*P* value
		Low (n=15)	Mixed (n=25)	High (n=30)		
**Organizational context, mean**						
	Size of the clinic^b^ (number of consultations per year)	2.5^#^	5.6^^^	4.7^*^	21.8	<.001
	Location of the clinic (1=rural/semirural, 0=urban)	0.3	0.3	0.5	0.7	.49
	Type of consultations offered (% without appointment)	21.3	28.3	30.2	0.9	.92
**Managerial context, mean**						
	Type of clinical governance (1=non-FMG^c^, 0=FMG)	0.5^^^	0.0^*^	0.1^*^	11.1	<.001
	Experience of the scheduling manager^d^	3.1	3.6	3.0	1.4	.25
**Implementation of MAS^e^ systems, mean**						
	iMAS^f^ system implemented (1=yes, 0=no)	0.27^*^	0.64^^^	0.73^^^	5.2	.008
	MAS system implemented (1=yes, 0=no)	0.47	0.52	0.47	0.1	.43
**Extended use of MAS systems, mean**						
	iMAS system functionalities used	0.2^#^	1.1^^^	0.9^^^	4.2	.02
	MAS system functionalities used	1.3	1.5	1.8	0.3	.76
**Integration of MAS systems, mean**						
	Integration of iMAS and MAS systems with the EMR^g,h^	0.9^*^	2.0^^^	2.2^^^	7.4	<.001
**Advanced accessibility, mean**						
	Advanced access scheduling principles applied	0.2^#^	1.5^*^	4.2^^^	118.3	<.001
**Availability of medical care, mean**						
	Scheduling performance^i^	3.0^*^	3.4^^^	3.6^^^	4.9	.01
	Patient attendance^j^	0.5^#^	2.3^^^	1.7^*^	23.8	<.001

^a^Within rows, different symbols (#, *, and ^) indicate significant (*P*<.05) pairwise differences between means (Tamhane T2 test).

^b^1: less than 5000, 2: 5000 to 9999, 3: 10,000 to 14,999, 4: 15,000 to 19,999, 5: 20,000 to 24,999, and 6: more than 25,000.

^c^FMG: family medicine group.

^d^1=less than 1 year, 2=1 to 3 years, 3=4 to 6 years, 4=7 to 9 years, and 5=more than 10 years.

^e^MAS: medical appointment scheduling.

^f^iMAS: interoperable medical appointment scheduling.

^g^iMAS system integration (no=0, manual=1, and automated=2) + MAS system integration (no=0, manual=1, and automated=2).

^h^EMR: electronic medical record.

^i^1=totally disagree, 2=rather disagree, 3=neither disagree nor agree, 4=rather agree, and 5=totally agree.

^j^0=less than 80%, 1=80% to 84%, 2=85% to 89%, 3=90% to 94%, and 4=95% or more.

To identify the technological, organizational, and managerial correlations of the sampled clinics’ medical care accessibility and availability, we contextualized the 3 profiles that emerged from the cluster analysis, as shown in Table 3. First, it can be noted that all clinics showed a similar organizational and managerial context except for low clinics, which tended to present a significantly lower number of consultations per year (between 5000 and 9999 compared to between 15,000 and 24,999). This implies smaller-sized clinics and a higher proportion of non-FMG clinics (approximately 50% compared to approximately 10% or less) in comparison to the clinics in the mixed and high groups. Second, the low clinics presented the lowest levels of iMAS system implementation and functionality usage, as well as the lowest levels of integration of iMAS and MAS systems with their EMR systems. In this regard, the results obtained for the mixed and high groups were comparable. There were no differences between them in terms of MAS system implementation. Third, concerning advanced access principles, the clinics in the high group showed a significantly higher level of application (4 principles applied on average out of a maximum of 6), followed by the mixed group (1.5 principles applied on average), which is still significantly higher than the low group level (0.2 principles applied on average). Finally, in terms of medical care availability, the low group presented the lowest results for scheduling performance and patient attendance. Although the high and mixed groups presented similar levels of scheduling performance, the mixed group had the highest patient attendance rate.

## Discussion

### Main Results

Using a mixed methods approach, this study surveyed a sample of medical clinics with the objective of gaining further knowledge on their assimilation of MAS systems. We also sought to gain insight into the performance benefits obtained by the clinics from their use of such systems. Notwithstanding the small sample size, the 6 experts we consulted stated that the results of this study accurately portrayed the situation in the family medicine clinics in Quebec with regard to medical appointment scheduling.

#### Clinics’ Assimilation of MAS Systems and Their Impacts on Accessibility of Care

The factors that contribute to the adoption of digital health technologies in general and MAS systems in particular are still unclear. An initial observation made in this study was that 17% of all clinics do not use any MAS system. A plausible explanation might be that some practices are more affected by the demographics of their patient population than others. In this line of thought, prior research found several patient-level barriers to the uptake of digital health technologies, including computer literacy, no or poor internet connection, and fear of using or lack of interest in technology [39,40]. Recent research also observed disparities in digital health technology adoption across sociodemographic subgroups, highlighting a persistent digital divide [41]. The rapid shift to digital care prompted by the COVID-19 pandemic demands research and action to ensure that underserved populations are not left behind [42].

Although MAS systems have been widely implemented in the surveyed clinics, these systems are only being used at a fraction of their full potential. Indeed, very few system functionalities, ranging from 1 to 2 on average, are being used. Clinics are mainly using those solutions that allow patients to manage their appointments via the internet and to automate appointment confirmations and reminders. Interestingly, the clinics that use more functionalities also tend to show improved performance in terms of accessibility of medical care. These findings are consistent with a previous study [13] showing that extended use of MAS systems in medical clinics is associated with improved care, as represented by an overall higher level of patient satisfaction and a lower level of missed appointments.

Another key observation is that despite considerable efforts to promote the use of advanced access principles throughout Quebec’s primary care clinics, our results showed that the 5 advanced access principles are being rather weakly applied. In fact, the 2 main principles applied in the sampled clinics included opening the physicians’ schedules over a “short” period of approximately 2 to 4 weeks, followed by balancing supply and demand, and incorporating interprofessional practice. A recently published study protocol [43] underscored the need for more detailed and representative data in this regard. Nevertheless, our study supports the idea that the greater the application of the advanced access principles, the higher the accessibility of medical services, and the better the clinics’ patient attendance rate and scheduling performance.

As observed in Figure 2, the results of our causal analysis also confirmed the proposition that integrating the MAS and EMR systems is a precondition to the successful assimilation of MAS systems in family medicine clinics. This integration provides a facilitating technological context for clinics seeking more integrated and extended usage of these systems [44]. Moreover, to explore the organizational and managerial factors that drive family medicine clinics to assimilate MAS systems, a cluster analysis was performed as a complement to the preceding causal analysis.

#### Characterization of Clinics Urgently Requiring Improvements Through MAS Systems

The cluster analysis allowed us to holistically characterize the family medicine clinics whose organizational performance urgently requires improvement in terms of the accessibility and availability of medical care they provide to their patients and to the general population. In this regard, clinics were categorized into 1 of 3 performance profiles: low, mixed, or high (accessibility and availability). When comparing how each group differs from the other two, our analysis showed that clinics in the low group had an organizational, managerial, and technological context that differed substantially from the mixed and high groups. These clinics may be characterized as “late implementers” with regard to their implementation of the iMAS system and as “beginners” (as opposed to “advanced users”) with regard to their assimilation of MAS systems. Low clinics also lagged behind in terms of MAS-EMR integration, irrespective of their interoperability, and their application of advanced access principles. In addition, they tended to be smaller in size and were not FMG clinics. Therefore, one may conclude that smaller clinics that are not FMGs are less likely to adopt and use an iMAS system or integrate their EMR into their MAS solution.

Although Quebec’s iMAS system has always been free for both clinics and patients, MAS systems are proprietary solutions available at a cost for clinics and sometimes for patients. It could be that smaller clinics, having less financial resources at their disposal, are less likely to adopt new technological solutions because of their greater relative cost. However, we found no statistical difference between the 3 groups of clinics in terms of MAS adoption and use. In addition to the iMAS solution being free, considerable efforts have been made by Quebec’s health care authorities to encourage clinics to adopt its centralized solution, including placing some “political” pressure on FMGs. In fact, we did find that a high proportion of FMGs had adopted Quebec’s iMAS system.

The experts we interviewed proposed additional explanations as to why the low group of clinics had a lower iMAS implementation rate. Importantly, the overall rate of approximately 60% was in line with the experts’ perceptions of the overall situation in family medicine clinics in Quebec. This could be explained in different ways. One is that the iMAS solution, although free, might be perceived by physicians as a means for the government to control how they manage their patient schedules. Irrespective of the validity of this perception, it appears to be real and widespread among general practitioners in Quebec. Physicians might also be reluctant to use an “imposed” MAS solution in a medical software market where other alternative solutions exist. It is also possible that such alternative solutions, albeit offered by the private sector, offer services and provide functionalities that are better suited to the needs of physicians and their clinics. Moreover, in Quebec, FMG clinics receive financial aid for supporting their digitalization that is based on their size. This might partially explain why larger FMG clinics tend to show a higher MAS implementation rate. According the experts’ opinions, smaller clinics prefer having experienced administrative personnel manage their physicians’ schedules, believing that they best know how to optimize each physician’s schedule based on their patients’ needs for care. Another possible explanation is that MAS systems provide true added value once the number of medical appointments reaches a certain threshold. This threshold may be the maximum number of daily appointments that a scheduling administrator can manage efficiently.

Since the clinics in the mixed group tended to be the largest and applied significantly fewer advanced access principles when compared to those in the high group, one could surmise from a strategic perspective that the larger clinics, operating at full capacity and being unable to cope with a growing demand for medical consultations, would want to limit rather than increase their accessibility, regardless of their assimilation of MAS systems. Although this last conjecture is in line with the surveyed experts’ opinions, it needs to be supported by further research on the strategic management of health IT in primary care settings [45].

### Contributions and Implications

The main contribution of this study is that it supports the idea that the adoption of MAS systems in family medicine clinics is by itself not sufficient to promote availability of care to patients and to the general population. Rather, it is the greater assimilation of the multiple functionalities of these systems that makes the difference in fostering patient attendance and enhancing scheduling performance. Whether the goal is to promote the application of advanced access principles in these clinics or their assimilation and the optimal use of MAS systems, further knowledge of the organizational, managerial, and, above all, technological factors that differentiate high-performing clinics from low-performing ones is required. This would allow clinic managers and physicians as well as consultants and governments to make better-informed plans and better-targeted recommendations on the development, promotion, adoption, and assimilation of MAS systems. Furthermore, this implies that it is mainly the family medicine clinics in the low group, and to a lesser extent in the mixed group, that should be targeted for improvement by national health authorities and health IT researchers and practitioners. Such efforts should support the following: (1) increased implementation of advanced access scheduling and (2) adoption and greater assimilation of MAS systems, particularly iMAS systems. This also implies that resources should be allocated to the low clinics for organizational learning purposes, and customized counseling and support should be offered throughout the MAS implementation process. As for the mixed clinics, counseling and support should lead them to optimize their use of MAS systems or iMAS solution. Another implication of our findings for future research lies in the renewed affirmation that in this digital health care era, the “assimilation” of medical systems [46]—rather than their mere adoption—and their “extended use” [47]—rather than mere use—are the key factors to achieving a better understanding of the impacts of such systems. Finally, this study makes a significant contribution toward research methodologies by analyzing the survey data through a combination of variable-oriented (SEM) and case-oriented (cluster analysis) approaches, thereby benefiting from the complementarity of these approaches to generate richer insights for researchers and practitioners [48].

### Limitations

The results of this study must be interpreted with care due to some inherent limitations. First, although 17% represents a good response rate for an online survey and all the surveyed experts confirmed that the study results matched their knowledge of family medicine clinics in Quebec, the limited sample size calls for some caution with respect to the generalizability of our findings. The second limitation pertains to how the performance of the sampled clinics was assessed. Ideally, it would have been preferable to survey more than 1 respondent per clinic to minimize common-method bias. Third, asking scheduling managers to evaluate their own performance may have somewhat biased the results; hence, a more objective assessment could have been made if iMAS, MAS, and EMR system data were available. Fourth, an intrinsic limitation of survey research is related to its cross-sectional nature, as true causality cannot be inferred. Future studies on the assimilation and impacts of MAS systems would thus warrant longitudinal designs.

### Conclusions

Notwithstanding the recent upheavals in health care brought about by the COVID-19 pandemic, access to and availability of primary care for populations remain a global issue. MAS systems are among the digital health solutions addressing these concerns by facilitating and optimizing the scheduling of medical appointments. As our data were collected in the very midst of the ongoing pandemic, our findings should be interpreted in light of the profound changes that have emerged in primary care settings in response to this crisis.

The main contribution of this study lies in its empirical demonstration that greater integration and assimilation of MAS systems in family medicine clinics lead to greater accessibility and availability of care for their patients and for the general population. Valuable insight has also been provided on how to identify clinics that would benefit the most from public- and private-sector initiatives to improve their efficiency and effectiveness as primary care providers for better primary care accessibility. Advancement of knowledge on this topic could benefit from similar studies carried out in contexts other than family medicine clinics, such as specialized or ambulatory care clinics.

## Abbreviations

EMR: electronic medical record
FMG: family medicine group
iMAS: interoperable medical appointment scheduling
IT: information technology
MAS: medical appointment scheduling
PLS: partial least squares
SEM: structural equation modeling
VIF: variance inflation factor
